# UV Fluorescence-Based Determination of Urinary Advanced Glycation End Products in Patients with Chronic Kidney Disease

**DOI:** 10.3390/diagnostics10010034

**Published:** 2020-01-09

**Authors:** Mieke Steenbeke, Sander De Bruyne, Elisabeth Van Aken, Griet Glorieux, Wim Van Biesen, Jonas Himpe, Gilles De Meester, Marijn Speeckaert, Joris Delanghe

**Affiliations:** 1 Department of Nephrology, Ghent University Hospital, 9000 Ghent, Belgium; Mieke.Steenbeke@uzgent.be (M.S.); Griet.Glorieux@ugent.be (G.G.); Wim.VanBiesen@ugent.be (W.V.B.); 2 Department of Laboratory Medicine, Clinical Chemistry, Ghent University Hospital, 9000 Ghent, Belgium; Sander.DeBruyne@uzgent.be (S.D.B.); Jonas.Himpe@ugent.be (J.H.); Gilles.DeMeester@ugent.be (G.D.M.); 3 Department of Ophthalmology, Sint-Elisabeth Ziekenhuis, 9620 Zottegem, Belgium; Elisabeth.VanAken@ugent.be; 4 Research Foundation Flanders, 1000 Brussels, Belgium; 5 Department of Diagnostic Sciences, Ghent University, 9000 Ghent, Belgium; Joris.Delanghe@ugent.be

**Keywords:** advanced glycation end products, chronic kidney disease, diabetes mellitus, urine, UV-fluorescence

## Abstract

Advanced glycation end products (AGEs) are a class of proteins or lipids that are non-enzymatically glycated and oxidized after contact with aldose sugars. The accumulation of AGEs results in carbonyl stress, which is characteristic for diabetes mellitus, uremia, atherosclerosis and vascular dysfunction. In recent decades, several innovative methods have been developed to measure the concentration of AGEs in blood or urine. In the present study, we evaluated the use of UV fluorescence as an alternative tool to detect urinary AGEs in four groups of well characterized chronic kidney disease (CKD) patients over a wide range of kidney insufficiency and in a group of healthy subjects. Using an excitation wavelength of 365 nm, the fluorescence spectra of urinary AGEs were recorded in the 400–620 nm emission range. When considering the emission peaks at 440 nm and 490 nm, a significantly higher AGE-specific fluorescence intensity was detected in CKD patients compared to healthy subjects (*p* < 0.0001 and *p* = 0.0001, respectively). The urinary creatinine adjusted fluorescence emission spectra in the group of CKD patients with diabetes mellitus were comparable with those of CKD patients without diabetes mellitus. Creatinine-adjusted fluorescence emission spectra were highest in CKD patients with proteinuria, moderate in CKD patients without proteinuria and lowest in healthy controls (*p* < 0.0001 at both emission wavelengths). In a multiple regression analysis, age, CRP and insulin treatment were predictors of fluorescence intensity at the emission wavelength of 440 nm. Age and insulin treatment were predictors at 490 nm. The presented method is a simple, cheap, alternative method to monitor the AGE-load in the CKD population.

## 1. Introduction

Advanced glycation end products (AGEs) are stable posttranslational modifications, formed by a non-enzymatic reaction of reducing sugars with free amino groups of proteins, lipids or nucleic acids [1,2,3,4]. This glycation process is known as the Maillard-reaction, which plays an important role in the chemical modification during food processing and the modification of proteins in the body. AGEs are involved in the natural ageing process, but also in a huge number of pathological conditions (e.g., diabetes mellitus, kidney failure, atherosclerosis, arterial hypertension, cataract and Alzheimer’s disease) [3,4,5,6]. AGEs can be present as a free form, as a single amino acid or as a free low-molecular-weight peptide, or bound to proteins, forming high-molecular-weight compounds [7]. 

Several methods have been developed to measure the concentration of AGEs in different human fluids: enzyme-linked immunosorbent assay (ELISA) [4,8,9], high-performance liquid chromatography (HPLC) [10], gas chromatography-mass spectrometry (GC-MS) [11], liquid chromatography-tandem mass spectrometry (LC-MS/MS) [12], immunoblotting [13], radio-receptor assay [14] and fluorescence spectroscopy [8,15]. The latter method is based on the principle that specific fluorescence is one of the main characteristics of AGEs. Several AGEs exhibit a characteristic fluorescence with an excitation wavelength in the range of 350–390 nm and an emission range of 400-620 nm [4,16,17,18]. Fluorescent AGEs are formed by the inter- and intra-molecular crosslinking of proteins [16], which is related to the initiation of diabetic complications [19]. Formation of crosslinks between proteins in the glomerular basal membrane may result in a permeability increase and to the development of albuminuria and consequently, of chronic kidney disease (CKD) [6,20]. A significant correlation has been observed between serum and urinary concentrations of AGEs [15].

The aim of the present study was to explore the possibilities of fluorescence spectrometry to detect urinary AGEs in well characterized CKD patient groups over a wide range of kidney failure (CKD stage 2–4, diabetics vs. non-diabetics, proteinuric vs. non-proteinuric kidney disease) in comparison with healthy individuals and to investigate the determining parameters of the AGE-specific fluorescence signal. 

## 2. Materials and Methods

### 2.1. Study Participants

The control group consisted of 31 healthy subjects (median age: 30.0 years, interquartile range (IQR: 25–53.5 years)), whereas the patient group consisted of 164 CKD patients (median age, IQR: 64.2–78.5 years) of the Department of Nephrology, Ghent University Hospital, Belgium. Table 1 describes the general characteristics of the 4 different groups of CKD patients and the healthy subjects. Group A consisted of CKD patients (CKD stage 2–4) with diabetes mellitus and proteinuria, group B of CKD patients (CKD stage 2–4) with diabetes mellitus and without proteinuria, group C of CKD patients (CKD stage 2–4) with proteinuria and without diabetes mellitus, and group D of CKD patients (CKD stage 2–4) without diabetes mellitus and proteinuria. In the group of diabetics, 13.7% had type 1 and 86.3% had type 2 diabetes mellitus with a mean glycated hemoglobin (HbA1c) of 7.2 ± 1.1%. The healthy subjects were recruited in group E. 

A spot urine sample was collected from all participants. The samples were centrifuged (754× *g*; 10 min at room temperature), aliquoted and stored at −80 °C. Samples were retrieved from a registered biobank (Biobank Kidney Ghent; EC2019/0216, Ghent, Belgium). Medications involved in renal function, AGE formation and inhibition of lipid peroxidation were collected. The approval of this study was granted by the Ethical committee of the Ghent University Hospital (EC/2010/033).

### 2.2. Fluorescence

Excitation-emission spectra of the urine samples were recorded using a FLAME-S-VIS-NIR-ES spectrometer (Ocean Optics, Largo, FL, USA) with a light source LLS-365 and an INSTMA-20 slit (slit width: 200 µm). One mL urine was transferred into a quartz cuvette with a 1 cm path length (Carl Zeiss, Oberkochen, Germany). The reflection probe (QR400-7-VIS-BX, premium 400 µm, VIS/NIR) (Ocean Optics, Largo, FL, USA) was positioned against the cuvette and a black background was used. The OceanView program (Ocean Optics, Largo, FL, USA) was set with an integration time of 10 ms and an average of 128 scans. Using an excitation wavelength of 365 nm, the fluorescence spectra of urinary AGEs were recorded at a 400–620 nm emission range. After background correction, the fluorescence signal of each urine sample was measured. Normalized fluorescence spectra were prepared by dividing the relative fluorescence intensity at each wavelength by the (maximum) relative fluorescence intensity at the (corresponding) peak wavelength. As the urinary concentration of AGEs depends on the urine volume, the relative fluorescence intensity (expressed in arbitrary units) was adjusted for the urinary creatinine concentration.

### 2.3. Routine Laboratory Parameters

The urinary albumin concentration was determined on a Behring Nephelometer analyzer II (Siemens, Marburg, Germany) by immunonephelometry, and the total urinary protein concentration was measured by the pyrogallol method using a photometric assay on the Alinity c (Abbott, Chicago, IL, USA). Proteinuria was expressed as the total urinary protein concentration/the urinary creatinine concentration ratio. The urinary creatinine concentration was detected by an alkaline picrate assay using the Cobas c 701 (Roche, Basel, Switzerland). The hemoglobin concentration was measured with a Sysmex XE-5000 (Sysmex Europe GmbH, Norderstedt, Germany). HbA1c was analyzed by ion exchange chromatography on the Tosoh HLV-723 G8 (Tosoh, Tokyo, Japan). C-reactive protein (CRP) and serum creatinine were determined by a photometric assay on an Alinity c (Abbott Laboratories, Chicago, IL, USA). The estimated glomerular filtration rate (eGFR) was calculated with the chronic kidney disease epidemiology collaboration (CKD-EPI) formula [21]. Serum total cholesterol, high-density lipoprotein (HDL)-cholesterol and triglyceride levels were measured using a photometric assay on an Alinity c (Abbott Laboratories, Chicago, IL, USA). Low-density lipoprotein (LDL)-cholesterol values were derived using the Friedewald formula [22].

### 2.4. Statistical Analysis

Median spectra of each group were obtained using the SIMCA version 15.0.2 (Sartorius Stedim Biotech, Malmö, Sweden). All data analysis was performed with MedCalc version 9.4.2.0 (MedCalc Software, Ostend, Belgium). Normality of distributions was assessed using the D’Agostino–Pearson test. In case of normal distribution, data were presented as mean ± standard deviation. The median fluorescence intensities of each group at 440 and 490 nm, with corresponding 25th and 75th percentiles (IQR), were determined for not normally distributed data. The Mann–Whitney U test was used to test the differences between the two groups. For comparing more than two groups, the Kruskal–Wallis test was applied. To investigate correlations between non-normal continuous variables, Spearman’s rho (ρ) was calculated. Multiple regression was carried out to study the influence of various parameters on the fluorescence intensity. 

## 3. Results

**Figure 1:**
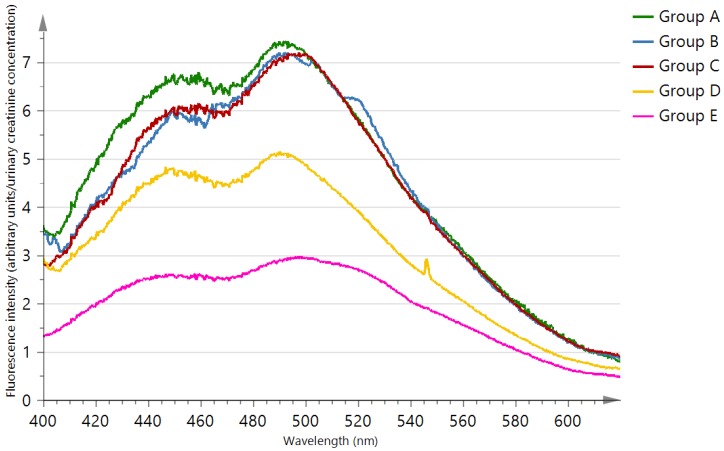
Median creatinine adjusted fluorescence spectra of chronic kidney disease (CKD) patients (group A: CKD patients with diabetes mellitus and proteinuria, group B: CKD patients with diabetes mellitus and without proteinuria, group C: CKD patients with proteinuria and without diabetes mellitus, group D: CKD patients without diabetes mellitus and proteinuria) and healthy subjects (group E).

Figure 1 shows the median urinary creatinine adjusted fluorescence emission spectra of the investigated groups. As illustrated in the figure, the fluorescence intensity was high in CKD patients with diabetes mellitus with or without proteinuria and with proteinuria alone (group A, B and C respectively), moderate in CKD patients without diabetes mellitus and without proteinuria (group D), and low in the healthy subjects (group E).

**Figure 2:**
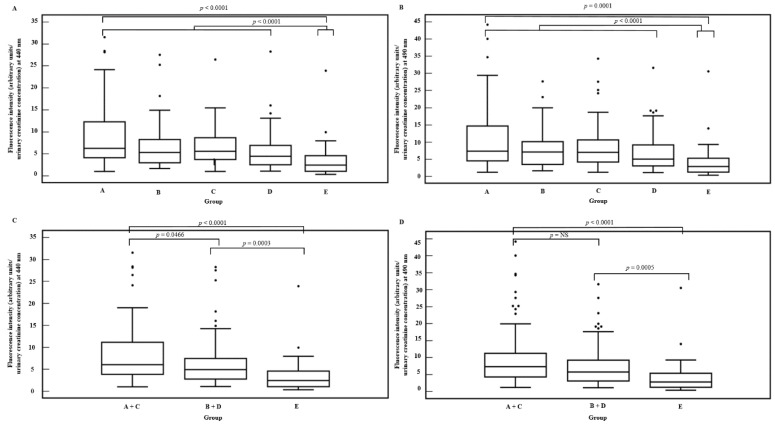
Creatinine adjusted fluorescence intensities at 440 nm (**A**) and 490 nm (**B**) for the group of chronic kidney disease (CKD) patients (group A: CKD patients with diabetes mellitus and proteinuria, group B: CKD patients with diabetes mellitus and without proteinuria, group C: CKD patients with proteinuria and without diabetes mellitus, group D: CKD patients without diabetes mellitus and without proteinuria) and healthy subjects (group E). Creatinine adjusted fluorescence intensities at 440 nm (**C**) and 490 nm (**D**) in CKD patients with proteinuria (group A + C), in CKD patients without proteinuria (group B + D) and in healthy subjects (group E).

As demonstrated in Figure 2, a significant difference in creatinine adjusted fluorescence emission spectra at 440 nm (Figure 2A) and 490 nm (Figure 2B) was observed between the groups (*p* < 0.0001 and *p* = 0.0001, respectively). Higher intensities were reported in CKD patients (group A + B + C + D) in comparison with healthy controls (*p* < 0.0001 at 440 nm and *p* < 0.0001 at 490 nm, respectively). The group of CKD patients with diabetes mellitus (group A + B) did not show significantly higher creatinine adjusted fluorescence emission spectra than those without diabetes mellitus (group C + D). Creatinine adjusted fluorescence emission spectra were highest in CKD patients with proteinuria (group A + C), moderate in CKD patients without proteinuria (group B + D) and lowest in healthy controls (group E) (*p* < 0.0001 at 440 nm and 490 nm). Higher creatinine adjusted fluorescence emission spectra at 440 nm were reported in CKD patients with proteinuria (group A + C) in comparison with those without proteinuria (group B + D) (Figure 2C) (*p* = 0.0466) and with healthy subjects (group E) (*p* < 0.0001). This result was not confirmed at 490 nm: only higher fluorescence intensities were seen in CKD patients with proteinuria (group A + C) in comparison with healthy subjects (group E) (*p* < 0.0001), but not in comparison with CKD patients without proteinuria (group B + D) (Figure 2D). Higher values were found in CKD patients without proteinuria (group B + D) in comparison with the healthy controls (group E) (*p* = 0.0003 at 440 nm and *p* = 0.0005 at 490 nm, respectively) (Figure 2C,D).

The correlation of the AGE-specific fluorescence intensity at 440 nm and 490 nm and the investigated parameters are shown in Table 2. 

No correlation was observed between the level of albuminuria or proteinuria and the fluorescence signal at 440 nm and 490 nm. No correlation was observed between HbA1c, serum levels of triglycerides, HDL-cholesterol and LDL-cholesterol, and the AGE-specific fluorescence intensity at both emission wavelengths. In a multiple regression analysis, age, eGFR, CRP and insulin treatment were predictors of the fluorescence intensity at the emission wavelength of 440 nm, whereas age, CRP and insulin treatment were predictors at 490 nm (Table 3). 

## 4. Discussion

In the present paper, we demonstrated that UV fluorescence is a fast, simple and affordable method for detecting urinary AGEs in CKD patients and this over a wide range of kidney function. The measurement of AGEs in urine with fluorescence spectroscopy has the advantage that it can be carried out in a non-invasive manner. Age, CRP and insulin treatment had a significant influence on the fluorescence intensity at 440 nm. Age and insulin treatment were predictors at 490 nm. 

At an excitation wavelength of 365 nm, the characteristic fluorescence signal of urinary AGEs was detected at emission wavelengths of 440 nm and 490 nm, which is in agreement with the literature [4,16,17,23,24]. A higher fluorescence intensity at both 440 nm and 490 nm was observed in CKD patients in comparison with healthy subjects, which could be explained by the stimulated formation and excretion of AGEs due to oxidative stress (e.g., uremia) and activation of the renin-angiotensin system [25]. AGEs contribute to the development of CKD by nonspecifically binding to and modification of basement membranes. By interaction with the receptor for AGE (RAGE), AGEs induce cellular responses, including the release of profibrogenic and proinflammatory cytokines, leading to interstitial fibrosis, glomerulosclerosis and tubular atrophy [26].

Comparable urinary AGE-specific fluorescence intensities were observed in diabetics and non-diabetics with CKD, which might be explained by a more prominent role in the formation of AGEs of oxidative stress in CKD patients in comparison with hyperglycemia and mitochondrial superoxide release in diabetics [26]. Oxidative stress is probably also the underlying reason why higher urinary AGE-specific fluorescence values were seen in CKD patients without proteinuria in comparison with healthy subjects. Future research should focus on measurement of specific biomarkers of oxidative stress (e.g., IsoPs, malondialdehyde, nitrotyrosine, S-glutathionylation, myeloperoxidase, oxidized low-density lipoprotein) to prove this hypothesis.

A positive correlation was observed between the urinary AGE fluorescence intensity and the CRP level, which is a marker of vascular inflammation. CKD patients have often increased levels of both CRP and AGEs. AGEs have been detected in the atherosclerotic plaques of patients with diabetes mellitus or CKD [27,28,29]. Due to their inflammatory effect, AGE indirectly enhance CRP expression via stimulation of IL-6 and IL-1 beta production [30]. CRP induces reactive oxygen species (ROS) formation, upregulates the expression of the receptor for AGEs and promotes endothelial progenitor cells sensitivity toward oxidative stress-mediated apoptosis. This contributes to the development and progression of atherosclerosis [31]. As the CKD population involved in this study was overweight/obese in comparison with the healthy population, the subclinical systemic inflammation characterized by an increased expression of IL-6 could also be a driver of increased CRP levels [32].

As confirmed in the present study, an accumulation of AGEs is observed during ageing as a physiological process, which is explained by the age-related increase in oxidative stress and less efficient repair mechanisms [33]. The accumulation of AGEs in many parts of the body results in significant changes in the metabolism and properties of the organs [34].

Finally, a significant correlation was observed between the fluorescence intensity at both emission wavelengths and insulin treatment. In the present study, 17 patients were treated with insulin (10 with diabetes mellitus type 1 and with proteinuria, 1 with diabetes mellitus type 2 and proteinuria, and 6 with diabetes mellitus type 2 and without proteinuria). Accumulation of AGEs may start early in the course of type 1 diabetes mellitus and may result in endothelial dysfunction and finally, diabetic nephropathy [35]. This could partly explain why a positive correlation was observed between the AGE-specific fluorescence intensities and insulin treatment. However, we could not exclude a direct effect or mechanism mediated by insulin treatment, which may also regulate or affect renal AGEs excretion. The described anti-AGE effect of antidiabetic and antihypertensive drugs as well as statins [36] could not be demonstrated in the present study population group.

UV AGE-specific fluorescence could potentially be used to monitor interventions to lower the concentration of AGEs: e.g., a low AGE diet [37]. AGEs in food generally have a low biological availability when ingested: only 10–30% of the total amount of AGEs in a meal is absorbed. A part of these exogenous AGEs is together with endogenous formed AGEs distributed to the tissues [5,26]. Of the portion absorbed AGEs, persons with a normal kidney function will excrete about one third into the urine within 48 h following intake. In end stage kidney disease, this percentage can drop to 5%. In healthy persons, the effect of dietary AGEs is negligible because of their low biological availability and good renal clearance [38,39]. AGE-rich food patterns can accelerate consequences of diabetes mellitus and the natural ageing process [40]. Control of dietary AGE-intake in persons with or without diabetes mellitus and/or kidney failure affects the AGE-concentration in blood [41]. In diabetics on a diet poor in AGEs, a reduction of the amount inflammatory mediators is observed [40,42]. In patients with kidney failure, restriction in dietary AGEs was associated with a decrease in serum AGE-concentration and CRP-values. An AGE-rich diet caused more micro- and macrovascular and endothelial damage than a low AGE diet in diabetes mellitus type 2 patients [39,43].

The measurement of the total fluorescence of all AGEs has some limitations as it does not allow to find out which compounds are responsible for the signal. As some AGEs (e.g., N-(Carboxymethyl)lysine (CML) and MG-derived hydroimidazolone (MG-H1)) show no fluorescence properties, the fluorescence intensity may underestimate the amount of urinary AGEs [18,44]. Moreover as mentioned, only small low-molecular-weight AGEs are excreted in the urine. Another drawback of the method is that occasionally, an overestimation of the fluorescence signal can be induced by nicotinamide adenine dinucleotide (phosphate) (NAD(P)H), flavin adenine dinucleotide (FAD), porphyrines and tryptophan [45].

In conclusion, UV fluorescence can detect the level of urinary AGEs in different stages of kidney insufficiency, which depends on age, eGFR and CRP. It is a simple, cheap alternative method in comparison with the most common methods to measure AGEs such as ELISA, HPLC and LC-MS/MS, which have the disadvantage of consuming both reagent and sample. 

## Figures and Tables

**Table 1:** General characteristics of the group of chronic kidney disease patients and the group of healthy subjects. Data are presented as median (interquartile range) (non-normal distribution) or mean ± standard deviation (normal distribution).

Characteristics	Chronic Kidney Disease Patients				Healthy Subjects	*p*-Value
	Group A	Group B	Group C	Group D		
**N**	46	27	45	46	31	
**Male/Female**	28/18	16/11	29/16	27/19	13/18	
**Age (years)**	68.5 (63–78)	72 (66.5–79.8)	68 (62–77.3)	70 (64–77)	30 (25–53.5)	<0.0001
**Diabetes mellitus**	+	+	-	-	-	
**Proteinuria**	+	-	+	-	-	
**eGFR (mL/min/1.73 m^2^)**	37.4 (29.4–42.3)	33.0 (26.0–39.9)	36.2 (29.0–46.7)	41.2 (28.7–51.7)	>90	<0.0001
**BMI (kg/m^2^)**	30.7 ± 4.8	30.8 ± 4.8	28.3 ± 4.2	27.4 ± 4.1	22.4 ± 3.3	<0.0001

Abbreviations: BMI, body mass index; eGFR, estimated glomerular filtration rate.

**Table 2:** Overview of the correlation between the advanced glycation end products (AGE)-specific fluorescence intensity at 440 nm and 490 nm and the investigated clinical parameters.

	Dependent Variable:Fluorescence Intensity			
	Ln(440 nm)		Ln(490 nm)	
Parameter	Correlation Coefficient	*p*-Value	Correlation Coefficient	*p*-Value
Age	0.3930	<0.0001	0.3806	<0.0001
BMI	0.1418	0.0480	0.1181	N.S.
Smoking	0.1728	0.0171	0.1938	0.0074
Ln(eGFR)	−0.3767	<0.0001	−0.3472	<0.0001
Ln(Urea)	0.1822	0.0199	0.1453	N.S.
Ln(CRP)	0.2416	0.0008	0.2210	0.0023
Hemoglobin	−0.1617	0.0392	−0.1489	N.S.
Treatment				
Aspirin	0.1840	0.0110	0.1582	0.0293
Peroral anticoagulant	0.1711	0.0183	0.1464	0.0439
ACE inhibitor or ARB	0.1541	0.0337	0.1401	N.S.
Beta blocker	0.2023	0.0051	0.1724	0.0174
Nondihydropyridine calcium	0.1834	0.0113	0.1524	0.0358
channel blocker				
Loop diuretic	0.2570	0.0003	0.2243	0.0019
Statin	0.2053	0.0045	0.1990	0.0059
Insulin	0.2793	0.0001	0.2386	0.0009
Vitamin D	0.1505	0.0382	0.1219	N.S.

Abbreviations: ACE, angiotensin converting enzyme; ARB, angiotensin II receptor blocker; CRP, C-reactive protein; eGFR, estimated glomerular filtration rate; N.S., not significant.

**Table 3:** Multiple regression model with the AGE-specific fluorescence intensity at the emission wavelengths of 440 nm and 490 nm as dependent variables.

Dependent Variable	Independent Variable	β (SE)	*p*-Value
Ln(Fluorescence intensity at emission wavelength 440 nm) R^2^ = 0.1970, *p* < 0.001	Age (years)	0.0107 (0.0046)	0.0206
	Ln(eGFR) (mL/min/1.73 m^2^)	−0.2565 (0.1429)	0.0743
	Ln(CRP) (mg/L)	0.1346 (0.0593)	0.0245
	Insulin treatment	0.2798 (0.0844)	0.0011
Ln(Fluorescence intensity at emission wavelength 490 nm) R^2^ = 0.1467, *p* < 0.001	Age (years)	0.0155 (0.0040)	0.0001
	Ln(CRP) (mg/L)	0.1166 (0.0632)	0.0667
	Insulin treatment	0.2664 (0.0880)	0.0028

Abbreviations: BMI, body mass index; CRP, C-reactive protein; eGFR, estimated glomerular filtration rate; R^2^, coefficient of determination; β, standardized regression coefficient; SE, standard error.
